# Effects of Inundation, Nutrient Availability and Plant Species Diversity on Fine Root Mass and Morphology Across a Saltmarsh Flooding Gradient

**DOI:** 10.3389/fpls.2018.00098

**Published:** 2018-02-06

**Authors:** Regine Redelstein, Thomas Dinter, Dietrich Hertel, Christoph Leuschner

**Affiliations:** ^1^Plant Ecology and Ecosystems Research, University of Goettingen, Goettingen, Germany; ^2^Soil Science of Temperate Ecosystems, University of Goettingen, Goettingen, Germany

**Keywords:** diversity effect, nutrient availability, root depth distribution, sediment texture, specific root area, tidal inundation gradient

## Abstract

Saltmarsh plants are exposed to multiple stresses including tidal inundation, salinity, wave action and sediment anoxia, which require specific root system adaptations to secure sufficient resource capture and firm anchorage in a temporary toxic environment. It is well known that many saltmarsh species develop large below-ground biomass (roots and rhizomes) but relations between fine roots, in particular, and the abiotic conditions in salt marshes are widely unknown. We studied fine root mass (<2 mm in diameter), fine root depth distribution and fine root morphology in three typical communities (*Spartina anglica*-dominated pioneer zone, *Atriplex portulacoides*-dominated lower marsh, *Elytrigia atherica*-dominated upper marsh) across elevational gradients in two tidal salt marshes of the German North Sea coast [a mostly sandy marsh on a barrier island (Spiekeroog), and a silty-clayey marsh on the mainland coast (Westerhever)]. Fine root mass in the 0–40 cm profile ranged between 750 and 2,500 g m^−2^ in all plots with maxima at both sites in the lower marsh with intermediate inundation frequency and highest plant species richness indicating an effect of biodiversity on fine root mass. Fine root mass and, even more, total fine root surface area (maximum 340 m^2^ m^−2^) were high compared to terrestrial grasslands, and were greater in the nutrient-poorer Spiekeroog marsh. Fine root density showed only a slight or no decrease toward 40 cm depth. We conclude that the standing fine root mass and morphology of these salt marshes is mainly under control of species identity and nutrient availability, but species richness is especially influential. The plants of the pioneer zone and lower marsh possess well adapted fine roots and large standing root masses despite the often water-saturated sediment.

## Introduction

The plants of temperate salt marshes are exposed to multiple stresses such as frequent flooding associated with salinity, temporary anoxia in the sediment, and possibly sulfide and manganese toxicity together with mechanical stress and sediment coverage (Leuschner and Ellenberg, 2017). Despite these constraints, some saltmarsh plants such as cordgrass (*Spartina* spp.) and sea purslane (*Atriplex portulacoides*) are known to be highly productive (Smith et al., 1979; Schubauer and Hopkinson, 1984; Bouchard and Lefeuvre, 2000). Many saltmarsh species develop extensive root systems and it has been found that plant biomass and productivity may be larger below- than above-ground in these environments (Valiela et al., 1976; Smith et al., 1979; Schubauer and Hopkinson, 1984; Groenendijk and Vinklievaart, 1987; Tripathee and Schaefer, 2015). For example, 50–90% of the productivity of *Spartina alterniflora* was found to be contributed by root and rhizome growth and turnover in a salt marsh in the eastern United States (Valiela et al., 1976; Darby and Turner, 2008). This suggests that a large part of the soil organic carbon contained in saltmarsh sediments is derived from roots, and below-ground productivity is an important factor in the carbon and nutrient cycles of these semi-aquatic ecosystems. Due to their short lifespan and rapid turnover, fine and very fine roots (diameters <2 mm) act as an important sink for carbohydrates supplied by photosynthesis (Jackson et al., 1997; Gill and Jackson, 2000). However, most studies on the saltmarsh below-ground compartment focus on the total (i.e., fine and coarse root, and rhizome) biomass, while only few studies have addressed the structure and dynamics of roots <2 mm in diameter, i.e., the fraction of the root system which likely is responsible for water and nutrient uptake.

Coastal salt marshes are extreme habitats, which require specific adaptations of the plants that colonize them. Species growing in the frequently inundated lower zone of the marsh have to cope with anoxia and reducing conditions in the soil. This environment may trigger the formation of aerenchyma in roots and rhizomes, which facilitate oxygen supply, and foster the development of strategies to exclude and excrete salt (Rozema et al., 1981, 1985). Root aerenchyma can increase the plant's capacity to detoxify potentially harmful ions such as S^2−^, Fe^2+^ or Mn^2+^ in the rhizosphere (Lee, 1999, 2003; Maricle and Lee, 2002). In the upper marsh, stress from inundation is less frequent, but plant growth may additionally be limited by nitrogen shortage (Valiela and Teal, 1974; Kiehl et al., 1997; Levine et al., 1998; van Wijnen and Bakker, 1999). The large below-ground biomass often found in salt marshes is thus not surprising, as it may be needed to secure nutrient and water acquisition, and to anchor the plants in a relatively unstable sediment. Root:shoot mass ratios exceeding unity are also found in other stressful environments such as nutrient-poor or dry grasslands and cold tundra ecosystems (Jackson et al., 1996; Leuschner et al., 2013).

Small-scale heterogeneity is a characteristic feature of many temperate saltmarsh ecosystems. Even minor elevation differences in the salt marsh may cause great spatial differences in inundation frequency, water level height, salinity and the degree of soil anoxia, and thus in the conditions for root growth in the sediment. This is also reflected in the zonation of saltmarsh communities (Bakker, 2014; Leuschner and Ellenberg, 2017), with salinity and tidal inundation as the main factors driving species distribution across the elevation gradient (Cooper, 1982; Armstrong et al., 1985; Rozema et al., 1985; Ungar, 1998).

Root system studies across elevation and water level gradients and in different sediment types should reflect the small-scale vegetation mosaic in salt marshes and may display the associated plant strategies to cope with varying environmental constraints. An example illustrating species differences is the study of Bouma et al. (2002) in a Dutch salt marsh, who found root longevity to be shorter in the highly competitive upper marsh grass *Elytrigia atherica* than in the more stress-tolerant grass *Spartina anglica* from the lowermost pioneer zone. Results of Steudel et al. (2011) and Ford et al. (2016) suggest that plant species richness, which can vary between one species per plot at the most stressful sites and more than 10 species at higher elevation, could also influence the root mass of saltmarsh communities, modifying the influence of abiotic factors. Also for other ecosystems it is known that plant diversity increases root biomass (Mommer et al., 2010; Mueller et al., 2013; Eisenhauer et al., 2017). Resource capture in more diverse communities may for example be enhanced by adjusting the depth distribution of roots between species (i.e., “complementarity effect”; Loreau and Hector, 2001; Cardinale et al., 2007; Mommer et al., 2010). Furthermore, the “selection effect” may increase biomass production in more diverse communities, when a very productive species dominates the biomass of the species mixture (Loreau and Hector, 2001).

In this study, we examined the variation in fine root mass, fine root depth distribution and fine root morphology across elevational gradients in two common types of North Sea tidal salt marshes, a barrier island marsh with mostly sandy sediment and a foreland salt marsh with silty-clayey sediment. Due to these differences in geomorphology, sites assumedly differ in nutrient availability and further soil properties, such as bulk density, enabling the investigation of these parameters on fine root traits. The variation in elevation and inundation frequency was addressed by transects reaching from the low-elevation pioneer zone with dominant *Spartina* stands with daily inundation to the high-elevation upper salt marsh with dominant *Elytrigia* swards that experience flooding only 4–8 times per month. We focused on fine roots <2 mm in diameter due to their relevance for resource uptake, while larger roots and rhizomes with primarily storage, conduction and anchorage function were not considered. We searched for those abiotic and biotic factors (inundation frequency, salinity, soil texture, soil nutrient and element content, species diversity), which exert the largest influence on fine root mass and morphology in the studied salt marshes. We tested the hypotheses that (i) fine root mass is on average greater in the sandy than the silty sediments due to higher nutrient availability in the latter, (ii) the decrease of rooting depth with increasing soil depth is less pronounced in the pioneer zone due to an assumed greater adaptive potential of the inhabiting species to the anoxic sediment conditions, (iii) the likely more stress-tolerant species of the frequently inundated pioneer zone have more robust roots with lower specific root area (SRA) and specific root length (SRL), but higher root tissue density (RTD) than the more competitive species of the upper marsh, and (iv) plant species richness has a positive effect on the root mass of the community.

## Materials and methods

### Study sites

**Figure 1:**
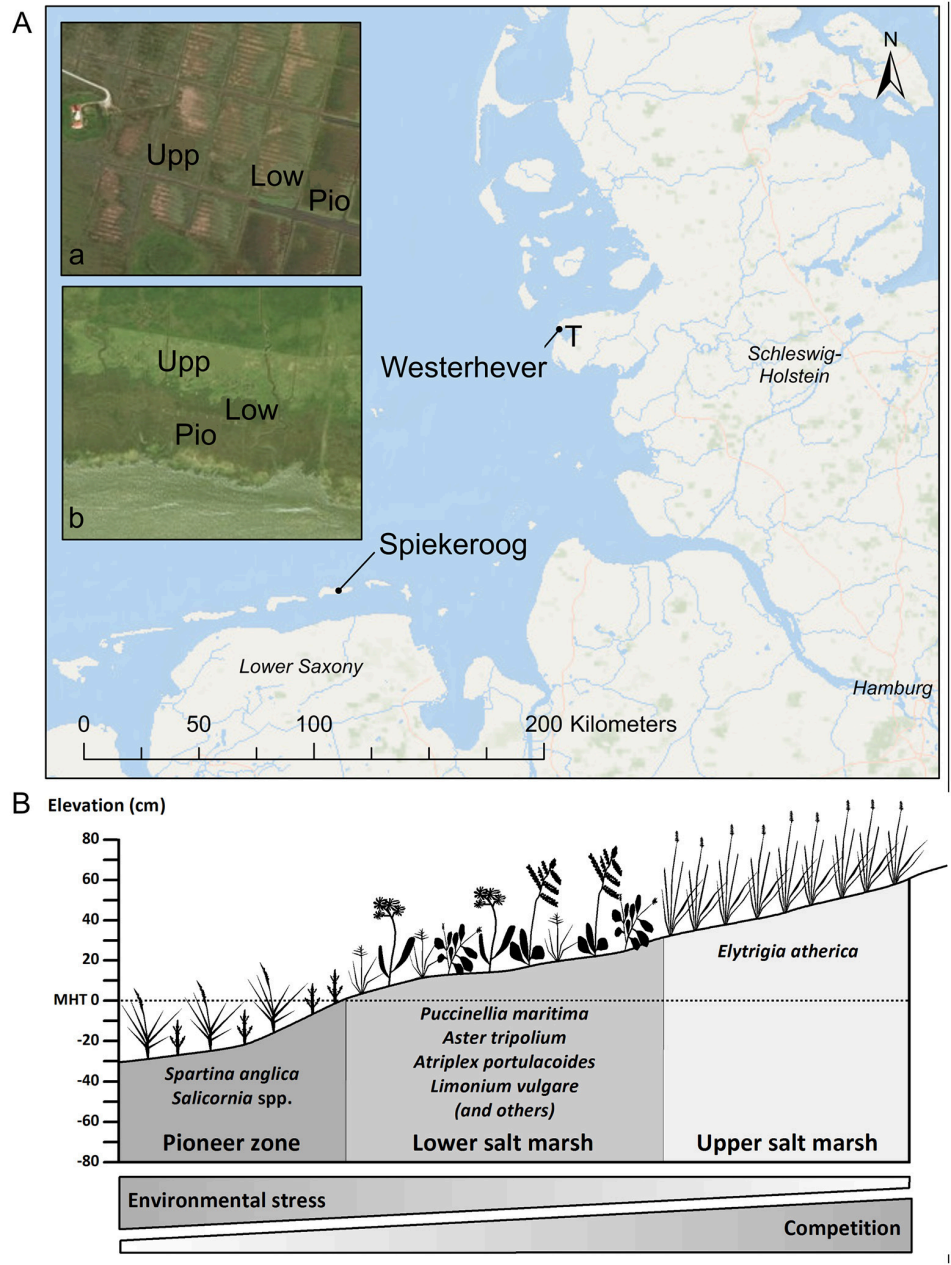
**(A)** Map of the German North Sea coast with the location of the two study sites Spiekeroog Island and Westerhever and location of the sampling plots in the three saltmarsh zones pioneer zone (Pio), lower salt marsh (Low), and upper salt marsh (Upp). Detail maps: Tümlau Bight in Westerhever (a) and south side of Spiekeroog Island (b). “‘T’ marks the location of the tide gauge station” from which water level data for Westerhever were obtained (Service layer Credits: Esri, DeLorme, GEBCO, NOAA NGDB, and other contributors, Source: Esri, DigitalGlobe, GeoEye, Earthstar Geographics, CNES/Airbus DS, USDA, USGS, AeroGRID, IGN, and the GIS User Community). **(B)** Zonation of a salt marsh with elevations relative to the mean high tide (MHT) water level and species typically inhabiting the three zones in a north-west European salt marsh.

Sampling was conducted in late September (i.e., toward the end of the growing season) at two saltmarsh sites of the German North Sea coast (Figure 1A): one site was located on the south coast of Spiekeroog Island (Lower Saxony, 53°45′44″N, 7°43′23″E), the second site was located in a salt marsh in the Tümlau Bay close to Westerhever (Schleswig-Holstein, 54°22′22″N, 8°38′47″E). Sites differ in terms of geomorphology: Spiekeroog is a barrier island with marshes on the leeward side developed on a base layer of sand on which a thin layer of silt has been deposited (Bakker, 2014). This marsh has developed naturally in the shelter of sand dunes and is not grazed by livestock. In contrast, the foreland salt marsh in Westerhever is located on the mainland coast on the seaward side of an artificial dike and has developed on a fine-grained sediment consisting of a thick (0.5–0.8 m) clayey silt layer (Peiter, 2004). This salt marsh was drained in the early twentieth century and has been intensively grazed until 1991 (Stock et al., 2005).

In both salt marshes, six plots of 2 m × 2 m size were installed in each of the three saltmarsh zones resulting in a total of 18 plots per site. Zones differed in elevation relative to sea level and thus inundation frequency and inhabiting saltmarsh communities (Figure 1B). The pioneer zone is inundated by most tides except the lowest neap tides and the dominant species is *Spartina anglica* C.E. Hubb., accompanied by *Salicornia stricta* Dumort.*, Suaeda maritima* (L.) Dumort., *Aster tripolium* L., and *Puccinellia maritima* (Huds.) Parl. The subsequent lower salt marsh, inundated less frequently only by spring tides, is the most species-rich community with high cover of *Atriplex portulacoides* L. and *Puccinellia maritima* and occurrence of *Cochlearia danica* L., *Suaeda maritima, Limonium vulgare* Mill., *Artemisia maritima* L., *Aster tripolium, Triglochin maritima* L., *Plantago maritima* L. s. str., *Salicornia europaea* L. s. str., *Spartina anglica* and *Spergularia media* (L.) C. Presl at lower frequency. The upper salt marsh is inundated only during very high spring tides and storm events and is dominated by *Elytrigia atherica* (Link) Kerguélen with a few individuals of *Atriplex prostrata* Boucher ex DC.

### Tidal inundation and soil properties

To measure water level variation and the frequency of tidal inundations of the plots, a RBR*duo* | TD wave sensor (RBR Ltd., Ontario, Canada) was installed on the tidal flat of Spiekeroog and its elevation determined relative to the height of the saltmarsh plots using a differential GPS. For the Westerhever site, data on water level fluctuation were provided from the nearby gauge station Tümlau AP (Figure 1A) by the Schleswig-Holstein Agency for Coastal Protection, National Park and Marine Conservation. The elevation of the Westerhever saltmarsh site is specified in Stock (2012). Inundation frequency and flooding duration of the three community types were calculated from the data on water level fluctuation and plot elevation.

From each plot, two soil samples for chemical analysis were taken, separated into the upper A_h_/A_i_-horizon (mineral horizon of the topsoil with accumulation of humus (h) or initial humus development in the pioneer zone (i);~0–20 cm depth) and the lower G_o_/G_r_-horizon (horizon affected by groundwater, oxidized (o) or reduced (r); ~ >20 cm depth) and combined to one pooled sample per soil horizon. For the analysis of grain size, an aliquot (5 g) of dried soil (105°C) was used. To account for organic substances, carbonates and iron-oxides, samples were treated with H_2_O_2_, HCl and Na-Dithionite prior to analysis. Particles >20 μm were separated using sieves of different mesh sizes, whereas particles <20 μm were separated by performing Atterberg cylinder analysis of sinking velocity. The remaining soil was dried at 40°C until constant weight and used for chemical analyses. Samples were sieved to 2 mm and ground in a ball mill (200 rpm for 5 min). One aliquot of a sample was digested with 65% HNO_3_ (Heinrichs et al., 1986) and total element contents (Al, Ca, Fe, K, Mg, Mn, Na, P, S) were determined using Inductively Coupled Plasma—Optical Emission Spectroscopy (ICP-OES, iCAP 6300 Duo View ICP Spectrometer, Thermo Fisher Scientific GmbH, Dreieich, Germany). From another aliquot, inorganic carbon was removed by treatment with 1 M HCl; this sample was dried at 50°C and analyzed for organic carbon (C_org_) and total nitrogen (N_t_) using a C/N elemental analyzer (Flash 2000, Thermo Fisher Scientific, Cambridge, UK). A third aliquot was used for determination of plant-available phosphorous according to Schüller (1969): 5 g of soil were added to 100 ml of calcium-acetate-lactate (CAL)-solution and shaken automatically for 90 min. Extracts were filtered into Falcon® Tubes, whereby the first 5 ml were discarded, and stored at 4°C until further analysis no longer than 24 h. Plant-available phosphorous was measured photometrically as a molybdate-complex using a microplate reader at 820 nm wavelength. For the analysis of mineral nitrogen (${\text{NO}}_{3}^{-}$ and ${\text{NH}}_{4}^{+}$), a further aliquot of fresh soil was immediately frozen after sampling. 20 ml of a 2.5 M K_2_SO_4_ solution were added to 5 g of fresh soil and thoroughly mixed for 30 s. Samples were shaken for 2 h on an overhead shaker and finally filtered through a Whatman® (No. 2) filter. Concentrations of ammonium and nitrate in the extracts were determined using a continuous flow injection colorimeter (Cenco/Skalar Instruments, Breda, The Netherlands).

### Sampling and processing of fine roots and aboveground biomass

For the inventory of fine root mass, samples were taken with a stainless steel corer (35 mm diameter, 400 mm length) on all plots to a depth of 40 cm. Soil cores were divided into depth intervals of 0–5 cm, 5–10 cm, 10–20 cm, and 20–40 cm and the samples were stored frozen until processing in the laboratory. The sediment material was washed over a sieve with 200 μm mesh size to separate roots from finest-grained sediment. Under a stereomicroscope the roots were further cleaned and separated from rhizomes and other organic material. Only fine roots <2 mm in diameter were considered for analysis. After the extraction of larger fine root fragments (>1 cm length), the remaining sediment with small rootlets was evenly distributed on a sheet of filter paper subdivided into 36 squares according to Hertel and Leuschner (2002). Six of the squares were randomly selected and all fine root fragments in the squares collected quantitatively under a binocular. The dry mass of these subsamples was extrapolated to the 36 squares to estimate the total mass of finest rootlets in the sample. In selected subsamples, we separated dead from living fine root material under the microscope to estimate the fraction of living fine roots. A non-turgid stele and periderm, the loss of the stele, or differing root color and elasticity were used as indicators of root death. Since the proportion of dead fine roots was <10% in all samples, we decided to refrain from distinguishing between dead and live roots in every sample in order to process a larger number of replicate samples. Thus, all data refer to total fine root mass which consists to >90% of biomass.

Root morphological traits were determined by scanning the extracted roots on a flatbed scanner. Total root length, root surface area, root volume and root diameter were measured using the software WinRhizo (Régent Instruments, Quebec, Canada). After scanning, roots were oven dried at 70°C for 72 h and weighed to determine dry mass. Specific root length (SRL, root length/dry weight), specific root area (SRA, root surface area/dry weight) and root tissue density (RTD, dry weight/root volume) were calculated from these measurements. Cumulative root surface area (Root Area Index, RAI) was calculated from SRA and fine root mass for a specific plot. For analyzing the C and N content of root mass, the dried roots were ground in a vibrating disc mill and a subsample of 5 mg was analyzed in a C/N elemental analyzer by gas chromatography (vario EL III; elementar, Hanau, Germany).

The percentage cover of all species in the 2 m × 2 m plots was estimated with the Londo scale to the next 10% (>10% cover) or 1% (<10% cover). Plant species diversity in the 4 m^2^ plots was expressed by Shannon's H. Aboveground biomass was sampled in all plots by randomly placing a square of 10 cm × 10 cm in the plot and cutting all plant stems directly at the soil surface inside the square. Biomass was separated from necromass, cleaned in the laboratory, dried at 70°C for 72 h and weighed.

### Statistical analysis

Statistical analyses were conducted using R 3.3.2 software (R Development Core Team, 2016). One-way Analysis of Variance (ANOVA) with Tukey's HSD test was applied for multiple comparisons between saltmarsh communities and sites (ANOVA and HSD-test, packages “car” and “agricolae,” respectively). Where normality of residuals and homoscedasticity were not given, values were log-transformed to meet these requirements. When assumption of normality could not be achieved, we used the Kruskal-Wallis test (kruskalmc, package “pgirmess”) for multiple comparisons between communities and sites. Welch's two sample *t*-test was used for comparisons between soil horizons. Step-wise multiple linear regressions with forward and backward variable selection were carried out with the “MASS” package to identify the best predictor variables for fine root mass. The initial model included total phosphorous content, mineral nitrogen content, Shannon's H, aboveground biomass, and the soil Na and S content; the final model was selected by means of minimum AIC. A significance level of *p* < 0.05 was used throughout. A principal components analysis (PCA) was conducted with the software CANOCO, version 5.02 (Biometris, the Netherlands) to analyze inter-relationships between fine root mass and morphology, and soil properties across the different saltmarsh communities and sites.

## Results

### Tidal inundation regime, soil properties, and plant species diversity

Due to the local tidal flat morphology, the pioneer zone plots in Spiekeroog were more frequently inundated than those in Westerhever (46 vs. 20 flooding events per month), whereas flooding events in the upper salt marsh occurred less frequently than in Westerhever (4 vs. 8 events per month; Table 1). The inundation frequency gradient from the pioneer zone to the upper salt marsh was thus steeper in the Spiekeroog transect.

**Table 1:** Summary of soil properties at the two study sites in the salt marshes of Spiekeroog Island and Westerhever on the German North Sea coast.

**Site**	**Spiekeroog**						**Westerhever**				
**Salt marsh zone**	**Pioneer zone**		**Lower salt marsh**		**Upper salt marsh**		**Pioneer zone**	**Lower salt marsh**		**Upper salt marsh**	
**No. of mean monthly flooding events (10.2014-09.2015)**	**46**		**16**		**4**		**20**	**16**		**8**	
**Sediment horizon**	**A_i_**	**G_o_/G_r_**	**A_h_**	**G_o_/G_r_**	**A_h_**	**G_o_/G_r_**	**A_i_**	**A_h_**	**G_o_/G_r_**	**A_h_**	**G_o_/G_r_**
Sand (>63 μm) fraction (%)	26.0 ± 1.9	98.2 ± 0.4	22.8 ± 4.9	97.5 ± 1.2	8.3 ± 1.6	92.4 ± 1.2	2.9 ± 0.0	7.1 ± 2.6	9.8 ± 1.8	17.7 ± 10.8	28.9 ± 5.5
Silt (2-63 μm) fraction (%)	41.2 ± 3.2	1.9 ± 0.4	41.6 ± 4.9	2.5 ± 1.2	54.1 ± 2.4	7.6 ± 1.2	62.5 ± 0.7	64.1 ± 1.9	62.4 ± 1.4	55.7 ± 4.4	49.7 ± 1.8
Clay (< 2 μm) fraction (%)	32.8 ± 1.4	0.0 ± 0.0	35.5 ± 5.2	0.0 ± 0.0	37.6 ± 4.0	0.0 ± 0.0	34.6 ± 0.7	28.8 ± 0.9	27.8 ± 0.5	26.6 ± 6.4	21.5 ± 3.7
Bulk density (g cm^−3^)	0.63 ± 0.01	1.62 ± 0.01	0.60 ± 0.04	1.68 ± 0.02	0.82 ± 0.08	1.63 ± 0.03	0.49 ± 0.01	0.71 ± 0.10	NA	0.62 ± 0.04	NA
pH (H_2_O) (min-max)	6.94–7.23	7.21–7.80	6.45–7.08	6.97–7.19	7.39–7.72	7.07–7.65	7.60–8.08	7.60–8.20	7.50–7.65	7.44–7.54	7.16–7.4
pH (KCl) (min-max)	6.87–7.05	6.81–7.78	6.23–7.23	6.48–6.74	6.99–7.19	6.67–7.39	7.07–7.20	7.10–7.34	7.04–7.19	6.92–7.14	6.88–7.13
Total nitrogen (%)	0.40 ± 0.03	NA	0.45 ± 0.12	NA	0.41 ± 0.08	NA	0.44 ± 0.01	0.42 ± 0.03	0.33 ± 0.03	0.33 ± 0.06	0.27 ± 0.05
Organic carbon (%)	3.20 ± 0.74	NA	4.40 ± 1.35	NA	3.54 ± 0.50	NA	5.25 ± 0.07	5.06 ± 0.27	3.89 ± 0.29	4.00 ± 0.69	3.24 ± 0.63
C:N ratio of soil	7.80 ± 1.38	NA	9.64 ± 0.37	NA	8.99 ± 0.62	NA	12.01 ± 0.16	12.09 ± 0.30	12.00 ± 0.31	12.31 ± 0.38	11.97 ± 0.54
Na (mg kg^−1^)	15, 632 ± 1, 064	3, 250 ± 173	12, 986 ± 1, 159	2, 702 ± 143	6, 789 ± 776	1, 453 ± 72	10, 829 ± 1, 606	8, 050 ± 2, 112	7, 805 ± 935	5, 473 ± 647	4, 706 ± 483
S (mg kg^−1^)	3, 557 ± 285	2, 239 ± 230	2, 284 ± 235	893 ± 260	1, 844 ± 176	215 ± 18	2, 591 ± 148	2, 861 ± 360	1, 572 ± 209	2, 311 ± 516	1, 453 ± 353
Plant-available P (mg kg^−1^)	84.46 ± 4.46	20.94	59.27 ± 6.85	59.73 ± 29.84	44.83 ± 4.83	10.50 ± 1.30	224.77 ± 34.37	98.47 ± 2.88	98.45 ± 5.25	94.77 ± 4.66	76.33 ± 6.78
Mineral N content (mg kg^−1^)	13.91 ± 2.89	1.70 ± 1.05	17.13 ± 3.40	0.88 ± 0.21	29.82 ± 4.02	1.21 ± 0.08	29.06 ± 5.58	21.10 ± 2.49	9.81 ± 1.14	33.64 ± 8.26	10.80 ± 5.12

*Given are means (±SE) of the three saltmarsh zones and the respective soil horizons. N_t_ and C_org_ in the Go/Gr horizons of Spiekeroog were close to the limit of quantification and could, thus, not be used for analysis. For statistical comparisons see Supplementary Material 1*.

*NA, data not available*.

The two study sites also differed with respect to sediment texture and chemistry (Table 1, Supplementary Material 1). The Spiekeroog site was characterized by a larger sand but smaller silt fraction in both the uppermost (A_h_) and lower sediment layers (G_o_/G_r_ horizon) compared to the generally finer textured Westerhever site (*p* < 0.05). An exception was the very silt- and clay-rich A_h_ horizon in the upper salt marsh of Spiekeroog. While the Westerhever sediment profiles were relatively uniform in terms of grain size distribution, the Spiekeroog profiles were clearly stratified with a distinct increase in the sand fraction from the A_h_ to the lower G_o_/G_r_ horizon (*p* < 0.05). This was reflected in a significant (*p* < 0.05) decrease in the mineral N and plant-available P content of the soil as well as in the Na and S contents toward the subsoil in Spiekeroog; this decrease was not as pronounced in Westerhever. At both sites, the grain size composition did not differ significantly between the pioneer, lower and upper saltmarsh zone. While the total N content of the sediment was similar across sites, the soil organic carbon content of the sediment and the related C:N ratio tended to be higher in Westerhever (significant in the topsoil of the pioneer zone and lower salt marsh, *p* < 0.05). While plant-available P was detected with significantly higher concentrations in the Westerhever sediments (*p* < 0.05), especially in the topsoil, the K_2_SO_4_-exchangeable nitrate and ammonium concentrations in Westerhever were higher in the deeper soil than in Spiekeroog (*p* < 0.05). The total Na and S contents were particularly high in the frequently inundated pioneer zone of Spiekeroog and decreased toward the upper salt marsh in both soil horizons (*p* < 0.05).

At both sites, the lower saltmarsh zones showed greatest plant species richness (10 species per plot at both sites) and Shannon diversity (H': 0.56 at Spiekeroog and 0.33 at Westerhever), whereas species richness and diversity in the upper salt marsh were lowest (2 species per plot; H': 0.02 at Spiekeroog and 0.03 at Westerhever). Mean species number in the pioneer zone plots was 4 (Spiekeroog) and 7 (Westerhever) and Shannon's H was 0.2 (Spiekeroog) and 0.27 (Westerhever) (Supplementary Material 2).

### Fine root and aboveground biomass and their dependence on environmental factors and plant diversity

**Figure 2:**
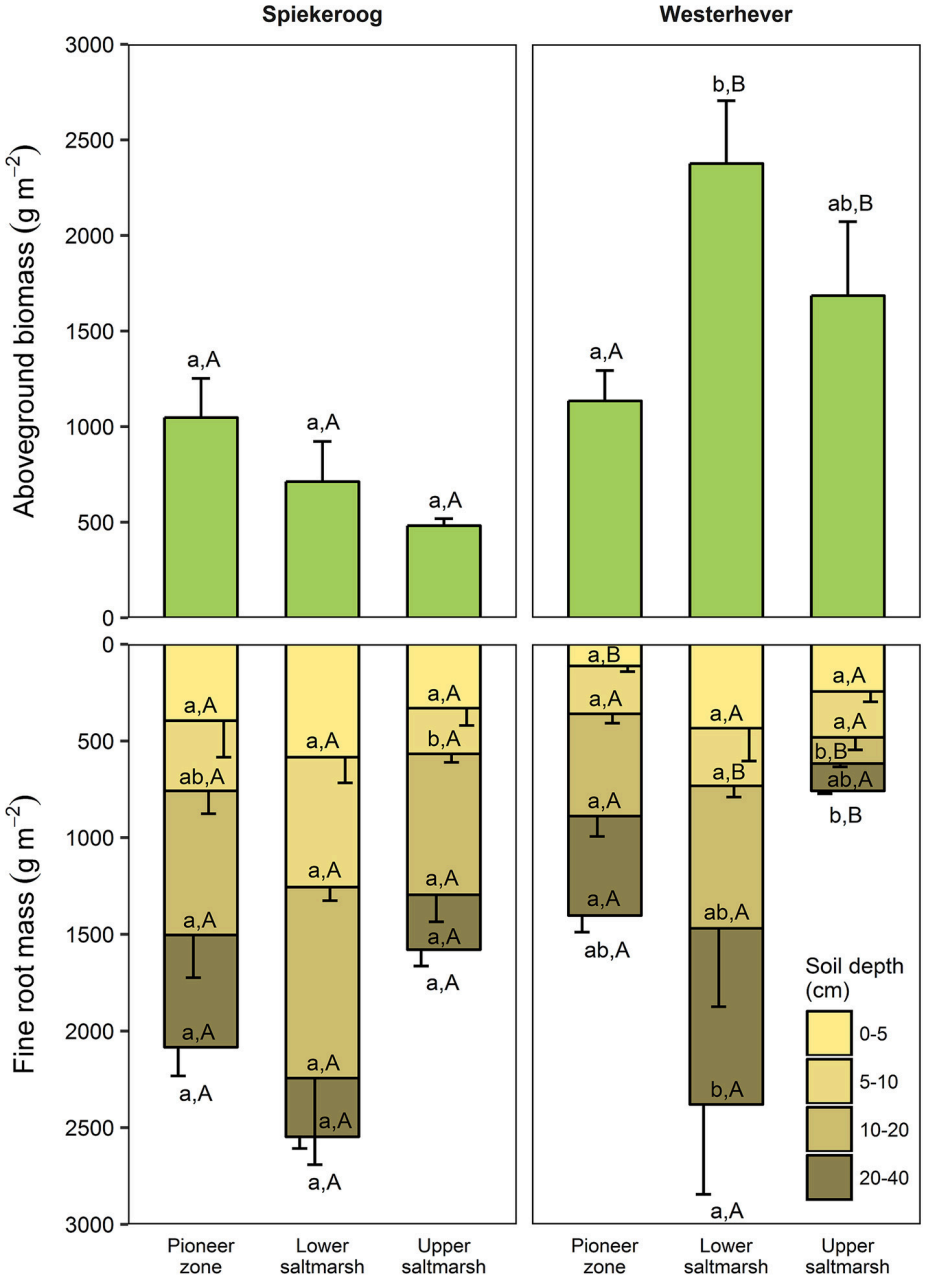
Aboveground biomass and fine root mass in the three saltmarsh communities at the two study sites (*n* = 6, means ± SE). Different letters indicate significant differences between communities at a site (a,b) or between the sites within a community type (A,B) (Kruskal-Wallis multiple comparison test, *p* < 0.05). Letters inside the bars indicate significant differences for different sediment horizons, letters below bars stand for the entire soil profile.

Total fine root mass (0–40 cm) was at both sites greatest in the lower salt marsh (2,547 g m^−2^ in Spiekeroog and 2,379 g m^−2^ in Westerhever) and smallest in the upper saltmarsh (1,579 g m^−2^ in Spiekeroog and 759 g m^−2^ in Westerhever) (Figure 2). However, this difference was only significant in Westerhever (*p* < 0.05). Aboveground biomass decreased in Spiekeroog from the pioneer zone (1,048 g m^−2^) to the upper salt marsh (481 g m^−2^) community (difference not significant), while it peaked in Westerhever in the lower salt marsh (2,376 g m^−2^), which was significantly greater than in the pioneer zone (1,134 g m^−2^, *p* < 0.05). Differences between the two sites in aboveground biomass in a given zone were larger than for fine root mass. In the Westerhever salt marsh, aboveground biomass was generally larger than in Spiekeroog on the sandy sediment (significant for the lower and upper salt marsh, *p* < 0.05) (Figure 2).

**Figure 3:**
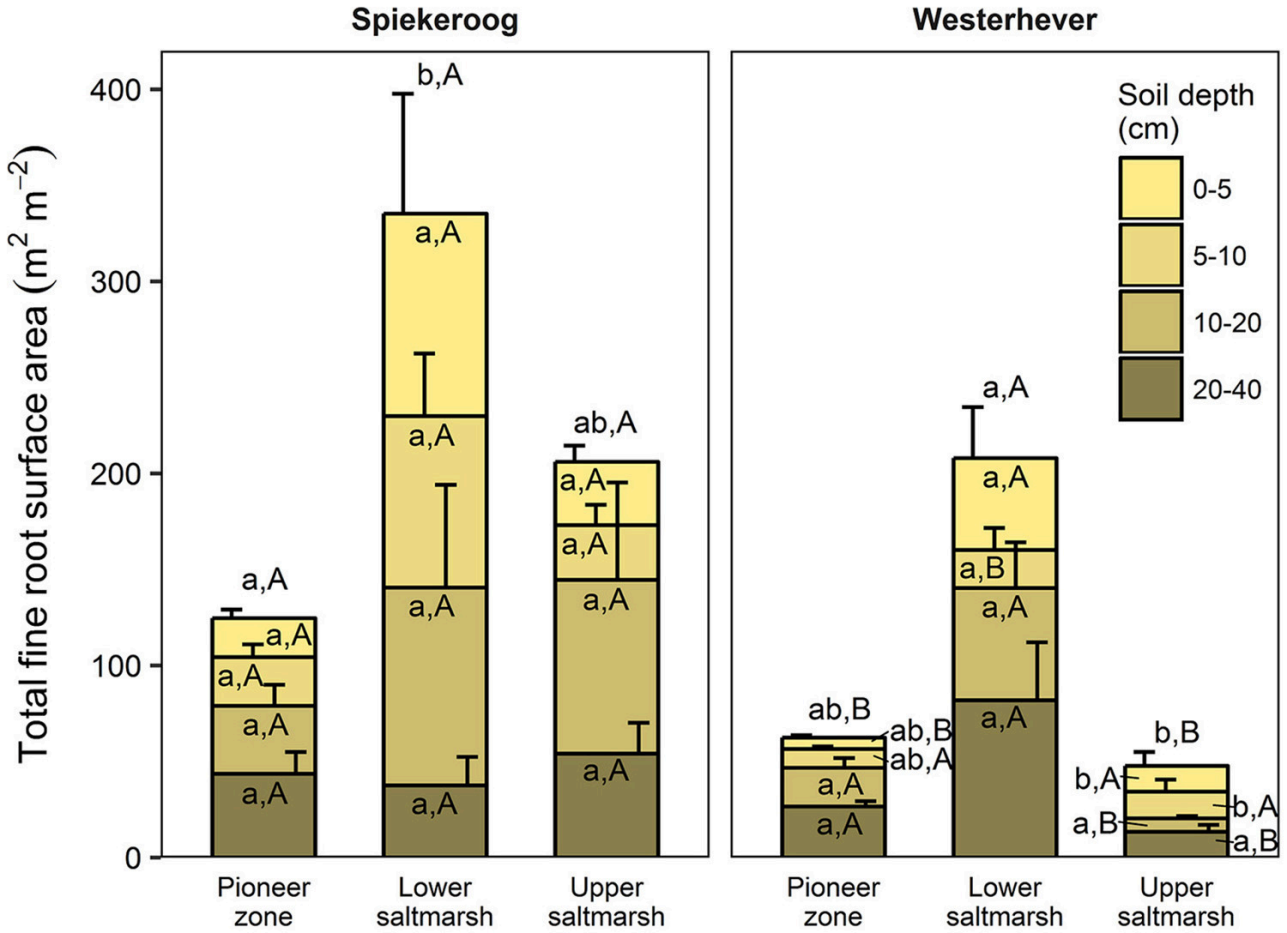
Cumulative fine root surface area per m^2^ ground area in the profile to 40 cm depth in the three saltmarsh communities at the study sites Spiekeroog and Westerhever (*n* = 6, means ± SE). Different letters indicate significant differences between the communities at a site (a,b) or between the two sites for a community type (A,B) according to Kruskal-Wallis multiple comparison tests (*p* < 0.05). Letters inside of the bars indicate significant differences for a given sediment horizon, letters above bars stand for the entire soil profile.

The ratio of fine root mass:aboveground biomass was in all communities at both sites greater than unity (except for the upper salt marsh in Westerhever), and reached a maximum of 4.8 in the lower salt marsh of Spiekeroog (Table 2). The ratio was generally greater in Spiekeroog than in Westerhever; this difference between sites was significant in the lower and upper salt marsh (*p* < 0.05) but diminished in the pioneer zone. Root area index (RAI, i.e., total fine root surface area) in the sediment to 40 cm was at both sites greatest in the lower salt marsh (335 and 205 m^2^ m^−2^ in Spiekeroog and Westerhever, respectively, Figure 3). In the pioneer zone and upper saltmarsh communities, RAI was significantly greater in Spiekeroog than in Westerhever (125 vs. 62 and 206 vs. 48 m^2^ m^−2^ in the pioneer zone and upper salt marsh, respectively; *p* < 0.05).

**Table 2:** Fine root mass:aboveground biomass ratio (median values) in the three saltmarsh zones at both study sites.

**Root:shoot biomass ratio**	**Pioneer zone**	**Lower salt marsh**	**Upper salt marsh**
Spiekeroog	1.85^a, A^	4.76^a, A^	3.52^a, A^
Westerhever	1.15^a, A^	1.20^a, B^	0.57^a, B^

*Different letters indicate significant differences between zones within a site (a) or between sites within a zone (A,B) analyzed by Kruskal-Wallis multiple comparisons (p < 0.05)*.

### Vertical fine root distribution

**Figure 4:**
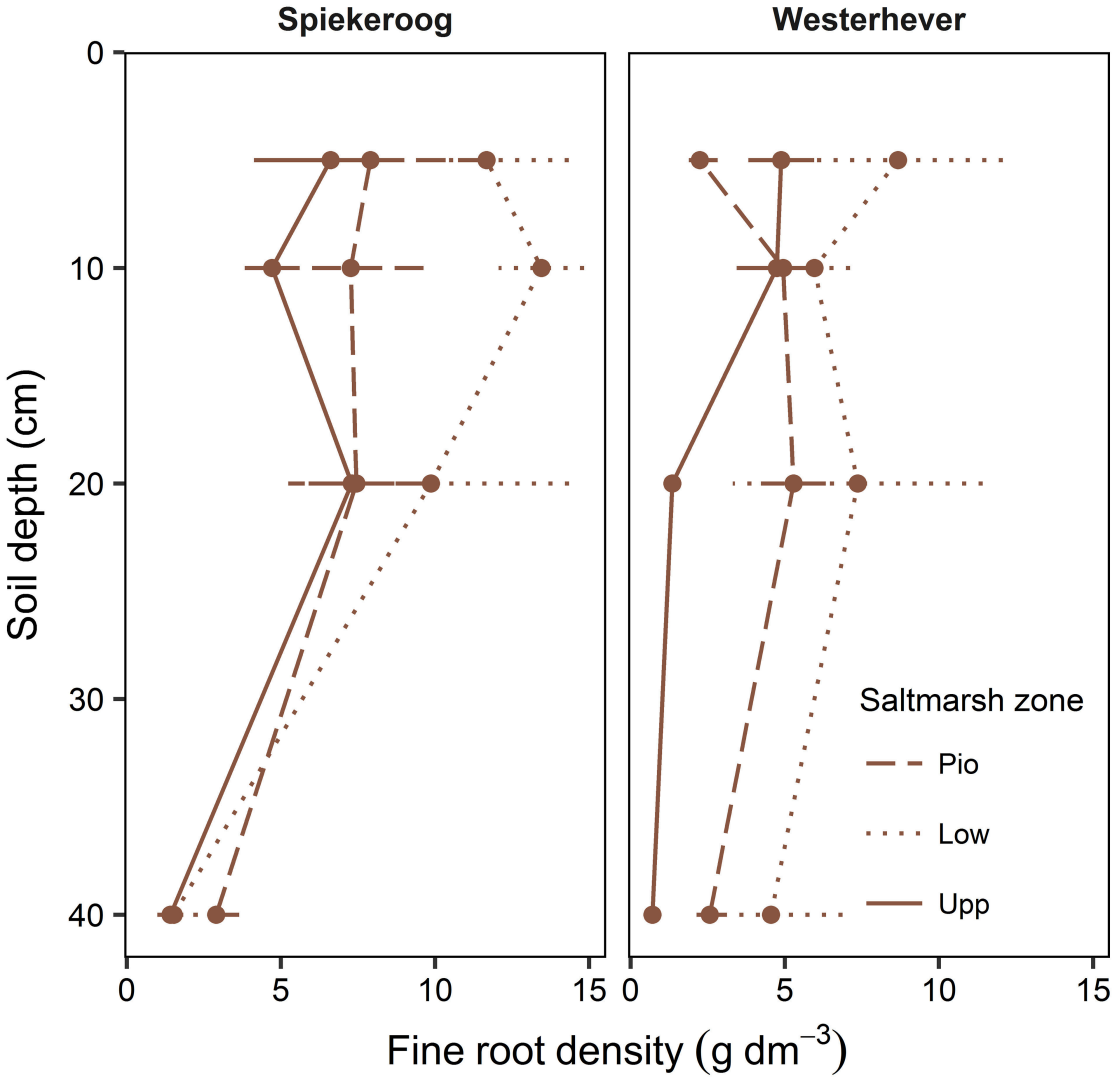
Change in fine root density (fine root mass per soil volume) with soil depth in the three saltmarsh communities (Pio, pioneer zone; Low, lower salt marsh; Upp, upper salt marsh) at the study sites Spiekeroog and Westerhever (*n* = 6, means ± SE).

The density of fine roots (root mass per soil volume) decreased with increasing depth in the sediment at both sites and in all communities (Figure 4). The decrease from the topsoil (0–5 cm) to the subsoil (20–40 cm) was, however, significant only in some cases (Westerhever: upper and lower salt marsh, Spiekeroog: upper salt marsh; Kruskal-Wallis multiple comparison test, *p* < 0.05, data not shown). At 10 cm soil depth, there was a remarkable difference in fine root density in the lower salt marsh with very high values in Spiekeroog (13.5 g dm^−3^) compared to Westerhever (6 g dm^−3^). At both sites, the pioneer zone held the lowest percentage of roots in the upper 20 cm of soil, i.e., the highest percentage of deep-reaching roots (significant for Westerhever, *p* < 0.05, Table 3).

**Table 3:** Percentage of fine root mass in the upper 20 cm of soil of the total soil profile (0–40 cm) (means ± SE, *n* = 6).

**Percentage root mass in upper 20 cm of soil**	**Pioneer zone**	**Lower salt marsh**	**Upper salt marsh**
Spiekeroog	69.48 ± 4.88^a, A^	86.16 ± 2.75^a, A^	82.88 ± 3.54^a, A^
Westerhever	64.08 ± 3.88^a, A^	66.05 ± 7.65^ab, B^	81.26 ± 1.71^b, A^

*Different letters indicate significant differences between zones within a site (a,b) or between sites within a zone (A,B) analyzed by Kruskal-Wallis multiple comparisons (p < 0.05)*.

### Relation between fine root mass and environmental factors and plant diversity

Stepwise multiple regression analysis with the variables total Na, P, S, and mineral N content in the sediment, Shannon diversity and aboveground biomass in the initial model revealed that Shannon diversity had a highly significant effect (*p* < 0.001) on fine root mass (Table 4). This effect had a higher explanatory power than that of mineral N content in the sediment (*p* < 0.01) and also of the Na content as a proxy of the inundation regime (*p* < 0.05). The prominent role of the influence of plant diversity disappeared when the root mass in the lower Go/Gr horizon was considered. Here, soil chemical parameters (Na and total P content) were the only influential factors (*p* < 0.01).

**Table 4:** Predictor variables for fine root mass identified by stepwise multiple regression analyses (forward and backward variable selection) grouped by study site and soil depth.

**Model variables**	**Estimate**	**se**	***t* value**	***p* value**
**BEST MODEL FIT: BOTH STUDY SITES, ALL SOIL DEPTHS**				
**(AIC = −92.75, *F* = 11.46, *df* = 3, 58, *p* < 0.001, *R*^2^ = 0.37)**				
(Intercept)	7.23	1.24 10^−1^	58.44	^***^
Na	2.49 10^−5^	1.23 10^−5^	2.02	^*^
mineral N	1.31 10^−2^	4.12 10^−3^	−3.18	^**^
Shannon-Wiener index, H	1.01	2.82 10^−1^	3.57	^***^
**BEST MODEL FIT: SPIEKEROOG, ALL SOIL DEPTHS**				
**(AIC = −79.18, *F* = 9.68, *df* = 2, 33, *p* < 0.001, *R*^2^ = 0.37)**				
(Intercept)	7.23	0.11	68.51	^***^
Shannon-Wiener index, H	0.80	0.20	4.03	^***^
aboveground biomass	0.00	0.00	1.62	n.s.
**BEST MODEL FIT: WESTERHEVER, ALL SOIL DEPTHS**				
**(AIC = −27.12, *F* = 6.10, *df* = 4, 21, *p* < 0.01, *R*^2^ = 0.54)**				
(Intercept)	6.15	3.43 10^−1^	17.92	^***^
Na	8.81 10^−5^	3.68 10^−5^	2.39	^*^
Shannon-Wiener index, H	1.80	7.53 10^−1^	2.39	^*^
aboveground biomass	2.93 10^−4^	1.39 10^−4^	2.11	^*^
total S	−2.2 10^−4^	1.43 10^−4^	−1.54	n.s.
**BEST MODEL FIT: BOTH STUDY SITES, A_h_ Horizon (AIC = −48.39**,				
***F* = 6.90, *df* = 3, 30, *p* < 0.01, *R*^2^ = 0.41)**				
(Intercept)	7.36	2.40 10^−1^	30.60	^***^
mineral N	−1.38 10^−2^	5.88 10^−3^	−2.35	^*^
Shannon-Wiener index, H	1.05	3.80 10^−1^	2.76	^**^
aboveground biomass	−1.69 10^−4^	9.00 10^−5^	−1.79	n.s.
**BEST MODEL FIT: BOTH STUDY SITES, G_o_/G_r_ Horizon (AIC = −13.29**,				
***F* = 4.05, *df* = 3, 24, *p* < 0.05, *R*^2^ = 0.34)**				
(Intercept)	5.05	0.28	18.343	^***^
Na	0.29 10^−3^	0.10 10^−3^	2.80	^**^
total P	−1.85 10^−3^	0.60 10^−3^	−3.06	^**^
aboveground biomass	0.32 10^−3^	0.24 10^−3^	1.34	n.s.

*n.s. p > 0.05*,

* *p < 0.05*,

** *p < 0.01*,

*** *p < 0.001*.

### Root morphological and chemical traits

**Figure 5:**
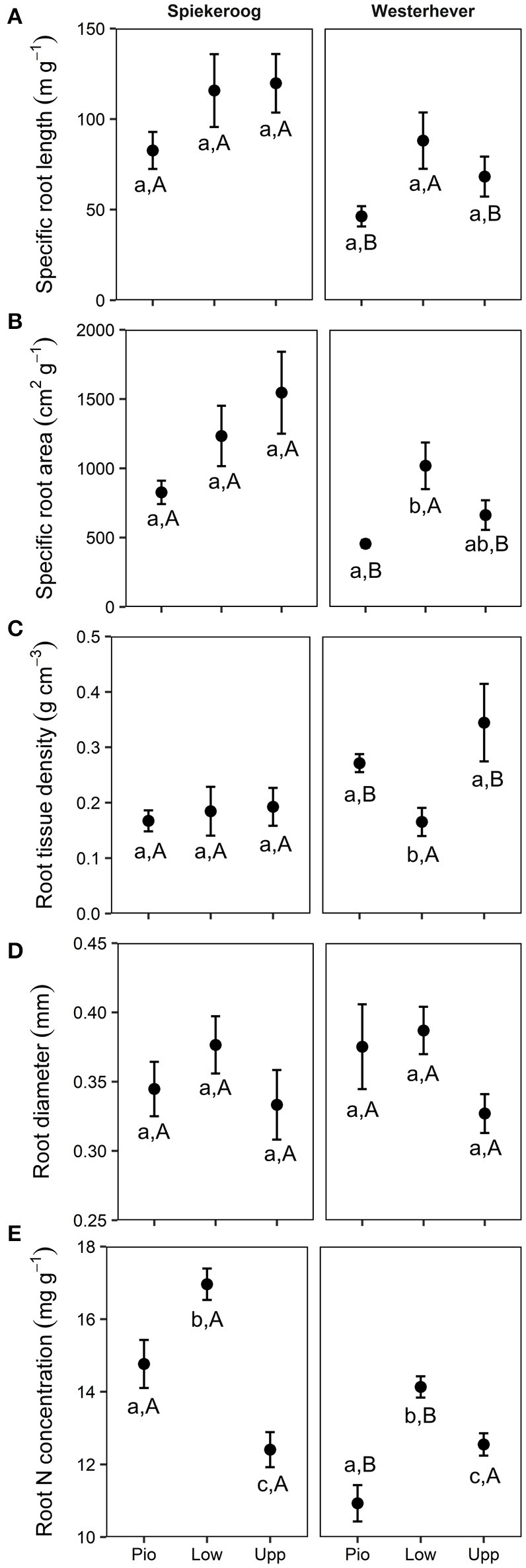
Means ± SE of five fine root morphological traits (**A**, Specific root length; **B**, Specific root area; **C**, Root tissue density; **D**, Root diameter; **E**, Root N concentration) in the three saltmarsh communities (Pio, pioneer zone; Low, lower salt marsh; Upp, upper salt marsh) at the study sites Spiekeroog and Westerhever (*n* = 24). Different letters indicate significant differences between study sites and saltmarsh communities according to one-way ANOVA with Tukey's *post-hoc* test (*p* < 0.05).

SRL and SRA ranged between 46 and 120 m g^−1^ and between 455 and 1,546 cm^2^ g^−1^, respectively, in all studied plots (Figure 5). Significant differences between zones (*p* < 0.05) were only found in Westerhever, where the highest SRL was found in the lower salt marsh. RTD ranged from 0.17 to 0.34 g cm^−3^ with particularly high values in the pioneer zone and upper salt marsh of Westerhever. Significant site differences existed for SRL, SRA (lower values in Westerhever) and RTD (higher values in Westerhever) in the pioneer zone and the upper saltmarsh community (*p* < 0.05). Root diameter was relatively uniform across the plots (0.3–0.4 mm) with no site- and community-specific differences. Root nitrogen concentrations differed between all three communities at both sites (*p* < 0.05). They were highest in the lower salt marsh of both sites (16.7 and 14.1 mg g^−1^ in Spiekeroog and Westerhever, respectively) and lowest in the upper salt marsh of Spiekeroog (12.4 mg g^−1^) and the pioneer zone of Westerhever (10.9 mg g^−1^). Particularly high values were found in the pioneer zone and lower salt marsh of Spiekeroog (significant difference to Westerhever, *p* < 0.05).

### Principal components analysis

**Figure 6:**
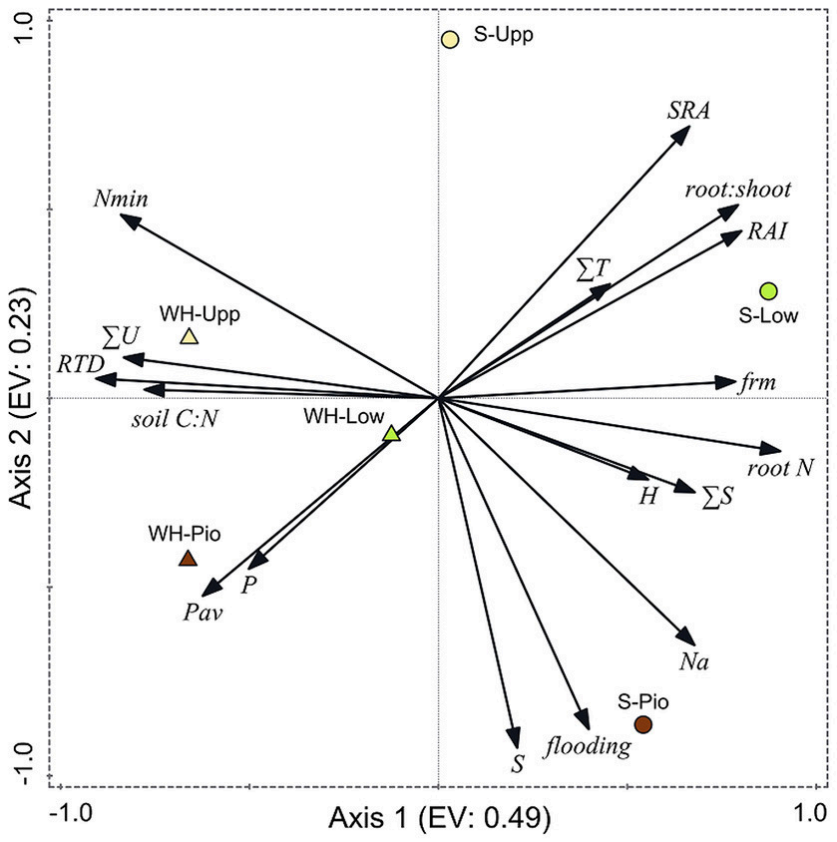
Plot showing the distribution of the three saltmarsh communities (Pio, pioneer zone; Low, lower salt marsh; Upp, upper salt marsh) of the two sites Spiekeroog (S) and Westerhever (WH) in relation to the PCA axes 1 and 2 (EV: eigenvalues of the axes) and their association with important soil and root properties (SRA, specific root area; ∑T, clay fraction; RAI, root area index; FRM, fine root mass; root N, root N concentration; ∑S, sand fraction; H, Shannon-Wiener index; Na, Na_t_ concentration in the sediment; flooding, no. of monthly flooding events; P, total P in the sediment; Pav, plant-available P in the sediment; RTD, root tissue density; ∑U, silt fraction; Nmin, mineral N concentration (${\text{NO}}_{3}^{-}$ and ${\text{NH}}_{4}^{+}$) in soil) in the upper soil horizon (A_h_). Vector length and angle are proportional to the direction and degree of their correlation with the plot ordination scores.

Ordination of the 36 plots with PCA based on the studied soil and root-related parameters confirmed the differentiation among the two study sites and three saltmarsh zones (Figure 6, Table 5). For the topsoil data (0–20 cm), the first axis (eigenvalue 0.48) separated the two study sites, and associated fine root mass, SRA, root:shoot ratio, RAI and root N concentration with the Spiekeroog site, whereas mineral N content, RTD and soil silt fraction were more closely associated with the Westerhever site. The second axis (eigenvalue 0.23) separated the three saltmarsh communities along a gradient of decreasing distance to the ocean. Mineral N as well as SRA were associated with this axis in the direction of the upper salt marsh, whereas on the opposite side, flooding frequency, S, Na and plant-available P contents of the soil were associated with this axis in the direction of the pioneer zone. The differences between the two study sites in terms of soil nutrient content in association with sediment texture were even clearer in the deeper sediment horizon (20–40 cm) than in the topsoil (Supplementary Material 3).

**Table 5:** Results of a Principal Components Analysis (PCA) on the differentiation of the three saltmarsh zones at both study sites with respect to fine root mass, root morphological traits, species diversity and soil properties of the A_h_-horizon.

	**Axis 1 (EV 0.4855)**	**Axis 2 (EV 0.2342)**
Fine root mass	**0.79** (0.62)	0.04 (0.62)
Fine root:shoot ratio	**0.79** (0.63)	**0.51** (0.89)
Specific root area	0.66 (0.44)	**0.72** (0.96)
Root tissue density	−**0.91** (0.82)	0.05 (0.82)
Root N concentration	**0.90** (0.82)	−0.14 (0.84)
Root surface area	**0.80** (0.64)	0.44 (0.84)
Total Na in soil	0.68 (0.46)	−**0.65** (0.89)
Total P in soil	−0.50 (0.25)	−0.45 (0.46)
Total S in soil	0.21 (0.04)	−**0.93** (0.90)
C:N ratio in soil	−**0.78** (0.60)	−0.02 (0.61)
Plant-available P	−0.62 (0.39)	−**0.52** (0.66)
Mineral N	−**0.84** (0.71)	0.49 (0.94)
∑ Sand	0.68 (0.46)	−0.25 (0.52)
∑ Silt	−**0.83** (0.69)	0.11 (0.71)
∑ Clay	0.45 (0.21)	0.30 (0.30)
H (Shannon-Wiener index)	0.56 (0.31)	−0.22 (0.36)
Flooding events	0.40 (0.16)	−**0.88** (0.93)

*Given are the loadings of the selected variables along the four explanatory axes. Numbers in brackets behind the axis indicate the eigenvalues (EV) of the axes. Numbers in bold mark the variables with closest correlation to the respective axis (cumulative fit values are given in brackets)*.

## Discussion

Our fine root inventory in three common saltmarsh communities at two geomorphologically different saltmarsh sites clearly showed differences in fine root mass between the three saltmarsh communities across the elevational gradient, but also between the two sites. At both study sites, the greatest fine root mass was recorded in the lower salt marsh, which correlated with highest plant diversity in these communities, where *Atriplex portulacoides* co-existed with about 10 other herb and grass species. We assume that in this lower saltmarsh community, a considerable diversity of below-ground space occupation strategies exists, as annuals (*Salicornia* spp.), small herbs (*Spergularia media*), tall herbs (e.g., *Aster tripolium, Triglochin maritima*), grasses (*Puccinellia maritima, Spartina anglica, Festuca rubra*) and dwarf shrubs (*Atriplex portulacoides*) share the rooted soil volume and likely partition space as explained by the “complementarity effect” (Loreau and Hector, 2001). In contrast, the upper saltmarsh community is nearly exclusively occupied by dense stands of *Elytrigia atherica*, and the pioneer zone is largely dominated by *Spartina anglica* with only low cover of additional grasses or herbs, which leaves less room for root space partitioning. This positive correlation between diversity and fine root mass in agreement with our hypothesis (iv) matches results from other root studies in salt marshes and other ecosystems and indicates an existing effect of biodiversity (Hooper et al., 2005; Cardinale et al., 2006). For example, Ford et al. (2016) found plant species richness to be a significant predictor of root biomass in a salt marsh in the UK. Similarly, in the mesocosm experiment of Sullivan et al. (2007) with various saltmarsh species, a significant increase in root biomass and also in the root:shoot ratio was recorded with increasing species diversity. These different reports suggest that plant diversity is a driver of fine root mass in temperate saltmarsh communities, in agreement with the findings in other non-saline grassland habitats (Mommer et al., 2010; Mueller et al., 2013; Eisenhauer et al., 2017; Weisser et al., 2017).

The lower saltmarsh zone plays a crucial role for sediment trapping and stabilization in the foreland bordering dykes and islands along the north-west European Wadden Sea coast. Our root data suggest that the relatively species-rich communities with abundant *A. portulacoides* are effective sediment stabilizers, since plant diversity was found to increase root density. This matches the observation that plant species richness enhances soil stability and erosion protection in salt marshes and other grasslands (Coops et al., 1996; Chen et al., 2012; Gould et al., 2016). Supporting previous studies (Chen et al., 2012), we suggest that *A. portulacoides* plays a role as key species in stabilizing sediment by its extensive root system in ungrazed saltmarsh sites.

The multiple regression analysis and the direct comparison of the three community types (zones) and two sediment types (sites) suggest that the principal abiotic factors salinity and inundation frequency only have a relatively small influence on fine root mass. The Na content of the sediment as a proxy of inundation duration showed a positive (though relatively weak) influence on root mass in the total sample (and in Westerhever), and root mass and root density reached a minimum in the upper marsh of both sites, where the exposure to salinity and inundation was lowest. This pattern is best explained by a dominant effect of plant species identity and species richness on community fine root mass, while effects of salinity (and anoxia) are diminished by effective adaptation of the species. It appears that productive species like *S. anglica* and *A. portulacoides* with specific adaptations to salinity and anoxia replace less adapted species in the lower marsh and pioneer zone, where they are capable of establishing large root systems. The photosynthesis of the C4 grass *Spartina* is rarely limited by salinity (Longstreth and Strain, 1977) and the species is capable of oxidizing sulfide in the roots and rhizosphere by means of effective O_2_ transport to the sediment through its aerenchyma (Lee, 1999, 2003; Maricle and Lee, 2002). Therefore, *S. anglica* is a highly productive species (exceeding 6,000 g d.m. m^−2^ yr^−1^; Long and Woolhouse, 1979) despite the mostly anoxic sediment. *A. portulacoides* is also highly tolerant of salinity (Redondo-Gómez et al., 2007) which is due to the accumulation of quaternary ammonium compounds for osmoregulation (Stewart and Lee, 1974; Rozema et al., 1985). The effective adaptation of the pioneer zone species (and also of the lower marsh zone taxa) to the adverse conditions in this environment is also reflected in a higher proportion of root mass (31–35%) in the subsoil (20–40 cm depth) of the pioneer zone compared to the upper salt marsh (17–19%). This is in accordance with our hypothesis (ii) in which we postulated a deeper root system in the more frequently inundated pioneer zone with well-adapted species. Interestingly, there was a great difference in fine root density between sites at 10 cm soil depth (Figure 4). It may be speculated that the very high fine root density in this soil depth found at Spiekeroog is related to the soil texture and associated lower plant-available P concentrations in the soil which may lead to a species-specific increase in fine root mass to compensate for limited P in the lower salt marsh. An earlier study by Strieckmann (1989, unpubl. data; Supplementary Material 4) also found no root density decrease down to 24 cm depth in other north German *S. anglica* marshes. In contrast, the fine root density decrease from the topsoil to 20–40 cm was significant in the upper salt marsh of both sites, indicating that *E. atherica* is more sensitive to waterlogging (Armstrong et al., 1985; Schröder et al., 2002; Veeneklaas et al., 2013) and thus develops a shallower distribution of fine roots.

Our data also suggest that higher nutrient availability tends to reduce fine root mass, as it was postulated in our first hypothesis. Fine root mass was higher by roughly 25%, and root area index even by about 50% in the sandy Spiekeroog plots compared to the silt- and clay-rich Westerhever plots. This may indicate that the plants in the sandy sediment need to produce larger root systems, with especially large surface area, to compensate for the generally lower concentrations of plant-available nutrients found in the sandy sediments of the Spiekeroog salt marsh. Sediment stability might also be responsible for differences in fine root mass: a higher root mass in the sandy Spiekeroog sediments could be required for plant anchorage, as fine-grained clay soils resist erosion better than sandy soils (van Eerdt, 1985; Allen, 1989). Since bulk density was higher at the sandy Spiekeroog site providing less air and water space, and less space for root proliferation, especially in the deeper soil horizon, it may be assumed that the need of fine roots for nutrient capture and plant stability exceeds the disadvantages for root growth in soil with higher bulk density, furthermore indicating the good adaptation of roots to the abiotic conditions.

Comparison of our fine root mass data to other studies on below-ground biomass in salt marshes is limited due to several reasons. First, it has to be kept in mind that we measured total fine root mass and not fine root biomass, even though our figures should be close to biomass according to our live:dead ratio assessment under the microscope in a number of subsamples. While a few authors explicitly sampled only fine root biomass, various studies do not state, whether live and dead roots were separated, and the term “biomass” is sometimes used for total root mass (biomass + necromass), which makes comparison to other studies difficult. Second, other authors used different criteria for defining root mass or biomass, sometimes including coarse roots and rhizomes as well, or retrieving root mass with sieves of greater mesh size than we did. This will increase or decrease root biomass figures, thus leading to deviating results. Groenendijk and Vinklievaart (1987) investigated total below-ground biomass in 0–60 cm in a Dutch salt marsh and obtained much higher profile totals than we did, which may only be partly explained by the lower profile depth investigated in our study (0–40 cm). Nevertheless, the same root mass distribution patterns along the saltmarsh elevational gradient became visible in the Dutch study: Greatest below-ground biomass totals were recorded in the *A. portulacoides*-dominated lower marsh (mean: 13,338 g m^−2^), while the lowest biomass occurred in the upper marsh dominated by *E. atherica* (mean: 7,763 g m^−2^). Comparison of our data from the *S. anglica* stands at our study sites (site means of 1,500–2,000 g m^−2^) with data from *Spartina alterniflora*-dominated salt marshes on the east coast of the United States indicates a similar root biomass average, but larger variation among sites (600–11,000 g m^−2^; Smith et al., 1979; Windham et al., 2003; Tripathee and Schaefer, 2015). Unpublished root biomass data from a grazed and an ungrazed salt marsh in Schleswig-Holstein (Germany) of Strieckmann (1989, unpublished) range between 1,000 and 5,000 g m^−2^ for the profile to 24 cm (Table 6, Supplementary Material 4). In contrast to our study, the greatest below-ground biomass in the ungrazed site was recorded in the plots dominated by *S. anglica* and *A. tripolium*, while the minimum (~1,500 g m^−2^) occurred in the *A. portulacoides* stands. Similar to our plots in Westerhever, the upper saltmarsh community with *E. atherica* dominance was the only stand with a root biomass:aboveground biomass ratio <1 (Table 6).

**Table 6:** Compilation of root mass and aboveground biomass data from North Sea salt marshes up to a sediment depth of 20 cm from this study and for two further sites investigated by Strieckmann (1989).

**Study site**	**Dominant plant species**	**Root mass (g m^−2^)**	**Aboveground biomass (g m^−2^)**	**Methodology**	**References**
Spiekeroog, Germany (ungrazed marsh)	*Spartina anglica*	1,503 ± 331	1, 048 ± 205	Size of sieve for root washing: 200 μm; Soil depth: 20 cm; only fine roots (dead and alive); sampling in September; mean ± se presented	This study
	*Atriplex portulacoides*	2,243 ± 484	712 ± 210		
	*Elytrigia atherica*	1,296 ± 111	481 ± 37		
Westerhever, Germany (ungrazed marsh)	*Spartina anglica*	888 ± 123	1, 134 ± 159		
	*Atriplex portulacoides*	1,469 ± 332	2, 376 ± 329		
	*Elytrigia atherica*	617 ± 46	1, 685 ± 387		
Oland, Germany (ungrazed marsh)	*Spartina anglica*	2,594	235 ± 146	Size of sieve for root washing: 315 μm; soil depth: 24 cm; no differentiation between root fractions; sampling in June; mean ± sd presented	Strieckmann, 1989, unpublished data
	*Spartina anglica* + *Aster tripolium*	5,156	783 ± 179		
	*Spartina anglica*	3,967	778 ± 184		
	*Atriplex portulacoides*	1,661	1, 191 ± 916		
	*Elytrigia atherica*	1,712	2, 178 ± 281		
Sönke-Nissen-Koog, Germany (grazed marsh)	*Puccinellia maritima*	3,496	518 ± 83		
	*Puccinellia maritima*	2,148	486 ± 164		
	*Spartina anglica*	2,743	499 ± 171		

The fact that we considered only fine roots (<2 mm in diameter) and not coarse roots and rhizomes, may explain differences in root biomass totals among different studies in *Spartina* marshes. For example, Darby and Turner (2008) found a mean root biomass of 753 g m^−2^, but a mean rhizome biomass of 1,952 g m^−2^ of *S. alterniflora* in a Louisiana salt marsh, matching findings of Schubauer and Hopkinson (1984). Data of total belowground biomass (including larger root diameters) are important for carbon cycle studies, but less informative when the below-ground absorptive surface of plants and communities is assessed. Several studies indicate that the standing fine root biomass (or mass) in salt marshes varies considerably with season (Groenendijk and Vinklievaart, 1987; Steinke et al., 1996; Darby and Turner, 2008), which may also explain differences between studies.

In our study, we did not distinguish between dead and live fine roots, as we found the proportion of dead fine roots to be low (<10%) in all inspected samples. Similar live:dead ratios were reported by Groenendijk and Vinklievaart (1987) in a Dutch salt marsh (<15% non-living roots), while the proportion of below-ground necromass was greater in certain North American salt marshes, exceeding root biomass (Valiela et al., 1976; Schubauer and Hopkinson, 1984; Darby and Turner, 2008). Apart from differences in the root diameter considered and likely variance in root mortality rates, this may be a consequence of slow root decomposition rates as was found in a Dutch salt marsh (Buth, 1987). Direct observation of fine root dynamics with rhizoscopes may be needed to unravel the causes of different root live:dead ratios in anoxic sediments.

A comparison of the fine root mass of salt marshes with that of other grassland or herbaceous communities indicates that the multi-stress conditions in this saline environment demand for high carbohydrate investment in below-ground organs by the plants. According to root mass data compiled by Leuschner and Ellenberg (2017), non-saline mesic to moist temperate grasslands have root masses in the topsoil (mostly 0–15 cm) of 500–2,000 g m^−2^ matching the root mass figures of our upper salt marsh, while being smaller than the root masses found in the lower marsh and the pioneer zone. Jackson et al. (1997) give a global fine root mass mean of 1,510 g m^−2^ for temperate grasslands, which is also lower than the root masses found in our lower salt marsh and in other *Spartina* stands in the northern hemisphere. Similarly, our root surface area totals (RAI values >300 m^2^ m^−2^) were several times larger than the mean RAI recorded for temperate grasslands by Jackson et al. (1997) (79.1 m^2^ m^−2^).

One might expect that the substantial variation in inundation frequency, sediment anoxia and salinity found in the different saltmarsh zones of Spiekeroog and Westerhever should lead to pronounced differences in fine root morphology in the different communities (hypothesis iii). Bouma et al. (2002) hypothesized that the saltmarsh plants of the pioneer zone have slower-growing, more stress-tolerant roots, while the plants of the upper salt marsh should be faster growing, which might result in a higher RTD of the former. We found elevated tissue densities in both the upper marsh and the pioneer zone of the Westerhever site, but no difference across the Spiekeroog gradient and thus no consistent RTD pattern in our study. Similarly, community differences in SRL and SRA were not consistent across the two elevational gradients; mean fine root diameter was remarkably constant in our samples. We conclude from these inconsistent patterns that fine root morphology is largely under the control of plant species and their specific adaptations to the adverse conditions (i.e., the formation of aerenchyma), while a more general pattern of root morphology did not emerge in the studied saltmarsh communities. This contradicts hypothesis (iii) but matches the findings of Bouma et al. (2001, 2002) of an only weak responsiveness of the root architecture of three halophytic grass species to nitrogen supply, inundation and oxygen content of the sediment and no relationship between root longevity and tissue density. Root N concentration peaked at both sites in the lower salt marsh, possibly reflecting species-specific N uptake patterns.

## Conclusions

Our study in two salt marshes with contrasting geomorphology shows that fine root mass is relatively high and fine root surface area large in comparison to grassland ecosystems in terrestrial habitats. The plants of the frequently inundated lower marsh and pioneer zone seem to be well adapted to this stressful environment, allowing some specialist species such as *Spartina* to establish a root system with deep penetration of the sediment for good anchorage and nutrient supply. These adaptations of characteristic species appear to control the fine root mass of the saltmarsh communities, overriding effects of environmental stress. As in other habitats with extreme environmental conditions, a higher number of plant species tends to increase root biomass and seems to promote soil exploration through root space partitioning by functionally different species. Studies on fine root dynamics and root function in terms of water and nutrient uptake are needed for a mechanistic understanding of the below-ground compartment of saltmarsh communities and its sensitivity to environmental change.

## Author contributions

CL, DH, and RR conceived and designed the research project. TD and RR performed field and laboratory work. DH, TD, and RR analyzed the data. CL and RR wrote the manuscript. All authors approved the final version of the manuscript.

### Conflict of interest statement

The authors declare that the research was conducted in the absence of any commercial or financial relationships that could be construed as a potential conflict of interest.
