# Supplementary material for: Effects of Inundation, Nutrient Availability and Plant Species Diversity on Fine Root Mass and Morphology Across a Saltmarsh Flooding Gradient
Source: Front Plant Sci. 2018 Feb 6;9:98. doi: 10.3389/fpls.2018.00098 (PMC5808222; doi:10.3389/fpls.2018.00098)
Supplement: Supplementary file 1 [file Table1.pdf]

Supplementary Material

Effects of inundation, nutrient availability and plant species diversity on fine root mass and morphology across a saltmarsh flooding gradient

Regine Redelstein*, Thomas Dinter, Dietrich Hertel, Christoph Leuschner

*** Correspondence:** Regine Redelstein: regine.redelstein@biologie.uni-goettingen.de

Supplementary Material 1: Results of statistical comparisons of soil properties

Table S1: Results of statistical comparisons of soil properties at the two study sites in the salt marshes of Spiekeroog Island and Westerhever on the German North Sea coast (data given in Table 1). Different letters mark statistically significant differences between zones (a,b), between sites (A,B), or between soil horizons (α, β) analyzed by Kruskal-Wallis multiple comparisons ($p < 0.05$) or Welch's two sample t-test for soil horizons ($p < 0.05$).

**Site**	**SPIEKEROOG**						**WESTERHEVER**				
**Salt marsh zone**	**Pioneer zone**		**Lower salt marsh**		**Upper salt marsh**		**Pioneer zone**	**Lower salt marsh**		**Upper salt marsh**	
**No. of mean monthly flooding events (10.2014-09.2015)**	46		16		4		20	16		8	
**Sediment horizon**	**A_i**	**G_o/G_r**	**A_h**	**G_o/G_r**	**A_h**	**G_o/G_r**	**A_i**	**A_h**	**G_o/G_r**	**A_h**	**G_o/G_r**
**Sand (> 63 µm) fraction (%)**	a,A,α	a,β	a,A,α	a,A,β	a,A,α	a,A,β	a,B	a,B,α	a,B,α	a,A,α	a,B,α
**Silt (2 - 63 µm) fraction (%)**	a,A,α	a,β	a,A,α	a,A,β	a,A,α	a,A,β	a,B	a,B,α	a,B,α	a,A,α	a,B,α
**Clay (< 2 µm) fraction (%)**	a,A,α	a,β	a,A,α	a,A,β	a,A,α	a,B,α	a,A	a,A,α	a,B,α	a,A,α	a,B,α
**Bulk density ($g\ cm^{-3}$)**	a,A,α	a,β	a,A,α	a,β	a,A,α	a,β	a,B	a,A	NA	a,B	NA
**pH (H_2O) (min-max)**	ab,A,α	a,β	a,A,α	b,A,β	b,A,α	ab,A,α	a,B	a,B,α	a,B,α	b,A,α	b,A,β
**pH (KCl) (min-max)**	ab,A,α	a,α	a,A,α	b,A,α	b,A,α	ab,A,α	a,B	a,B,α	a,B,β	b,A,α	a,A,α
**Total nitrogen (%)**	a,A	NA	a,A	NA	a,A	NA	a,A	a,A,α	a,α	a,A,α	a,α
**Organic carbon (%)**	a,A	NA	a,A	NA	a,A	NA	a,B	a,A,α	a,β	a,A,α	a,α
**C:N ratio of soil**	a,A	NA	a,A	NA	a,A	NA	a,B	a,B,α	a,α	a,A,α	a,α
**Na ($mg\ kg^{-1}$)**	a,A,α	a,β	ab,A,α	ab,A,β	b,A,α	b,A,β	a,B	a,A,α	a,B,α	a,A,α	b,B,α
**S ($mg\ kg^{-1}$)**	a,A,α	a,β	ab,A,α	ab,A,β	b,A,α	a,A,β	a,B	a,A,α	a,A,β	a,A,α	a,B,α
**plant-available P ($mg\ kg^{-1}$)**	a,A,α	NA	ab,A,α	a,A,α	b,A,α	a,A,β	a,B	a,B,α	a,A,α	a,B,α	a,B,α
**mineral N content ($mg\ kg^{-1}$)**	a,A,α	a,β	a,A,α	a,A,β	a,A,α	a,A,β	a,A	a,A,α	a,B,β	a,A,α	a,B,α

NA: data not available
