# Supplementary material for: Effects of Inundation, Nutrient Availability and Plant Species Diversity on Fine Root Mass and Morphology Across a Saltmarsh Flooding Gradient
Source: Front Plant Sci. 2018 Feb 6;9:98. doi: 10.3389/fpls.2018.00098 (PMC5808222; doi:10.3389/fpls.2018.00098)
Supplement: Supplementary file 2 [file Table2.pdf]

Supplementary Material

Effects of inundation, nutrient availability and plant species diversity on fine root mass and morphology across a saltmarsh flooding gradient

Regine Redelstein*, Thomas Dinter, Dietrich Hertel, Christoph Leuschner

*** Correspondence:** Regine Redelstein: regine.redelstein@biologie.uni-goettingen.de

Supplementary Material 2: Vegetation surveys of the sampling plots

Table S2.1: Vegetation cover estimated using the LONDO scale in the six replicate sampling plots of 2 x 2 m at the Westerhever site in the three saltmarsh zones (“Pio” = pioneer zone, “Low” = lower salt marsh, “Upp” = upper salt marsh).

Plot	Pio1	Pio2	Pio3	Pio4	Pio5	Pio6	Low1	Low2	Low3	Low4	Low5	Low6	Upp1	Upp2	Upp3	Upp4	Upp5	Upp6
Elytrigia atherica								0.4	0.1	0.1	0.2	0.1	10	10	10	10	10	10
Atriplex portulacoides	1	1	1	1	0.4	1	8	9	9	9	8	9						0.1
Spartina anglica	9	9	9	9	9	9	0.1	0.1	0.1	0.1	0.1	0.1						
Puccinellia maritima		0.2		0.1	0.2	0.2	0.4	1	0.4	0.2	0.2	0.2						
Suaeda maritima	0.1	0.1	0.1	0.1	0.1	0.2	0.4	0.1	0.1	0.1	0.2	0.1						
Triglochin maritima	0.2	0.1	0.1	0.1		0.1	10	0.1	0.1		0.2	0.1						
Aster tripolium		0.1	0.1	0.1	0.1	0.1	0.2	0.1	0.1	0.1	0.1	0.1						
Limonium vulgare			0.1				0.2	0.1	0.2	0.1	0.1	0.1						
Salicornia stricta	0.1	0.1	0.1			0.1	0.1			0.1	0.1							
Atriplex prostrata													0.1	0.1	0.1	0.1	0.1	0.1
Cochlearia danica							0.2	0.1	0.1	0.1	0.2							
Atriplex littoralis			0.1	0.1	0.1	0.1												
Spergularia media							0.1		0.1	0.1								
Plantago maritima								0.1	0.2									
Artemisia maritima											0.4							

Table S2.2: Vegetation cover estimated using the LONDO scale in the six replicate sampling plots of 2 x 2 m at the Spiekeroog site in the three saltmarsh zones (“Pio” = pioneer zone, “Low” = lower salt marsh, “Upp” = upper salt marsh).

**Plot**	**Pio1**	**Pio2**	**Pio3**	**Pio4**	**Pio5**	**Pio6**	**Low1**	**Low2**	**Low3**	**Low4**	**Low5**	**Low6**	**Upp1**	**Upp2**	**Upp3**	**Upp4**	**Upp5**	**Upp6**
Elytrigia atherica									0.1				10	10	10	10	10	10
Spartina anglica	6	9	9	9	9	9	0.1	0.1	0.1	0.1								
Atriplex portulacoides	2						8	2	2	6	6	1						
Puccinellia maritima					0.1	0.1	0.4	6	3	2	3	3						
Suaeda maritima	0.1	0.1			0.1	0.1	1	1	2	1	2	0.2						
Salicornia stricta	1	0.4	0.4	0.4	1	0.4	0.1	0.1	0.1	0.1	0.1	0.1						
Artemisia maritima							0.1		0.2	0.4	0.2	3						
Limonium vulgare						0.1	0.2	0.2	0.2	0.1	0.4	0.2						
Aster tripolium	0.1				0.1	0.1	0.1	0.1	0.1	0.1	0.1	0.1						
Spergularia media							0.1	0.1	0.1	0.1	0.1							
Triglochin maritima								0.1	0.1	0.1	0.1	0.1						
Atriplex prostrata													0.1	0.1		0.1	0.1	
Festuca rubra												2						
Plantago maritima										0.1								
