# Supplementary material for: Effects of Inundation, Nutrient Availability and Plant Species Diversity on Fine Root Mass and Morphology Across a Saltmarsh Flooding Gradient
Source: Front Plant Sci. 2018 Feb 6;9:98. doi: 10.3389/fpls.2018.00098 (PMC5808222; doi:10.3389/fpls.2018.00098)
Supplement: Supplementary file 3 [file DataSheet1.pdf]

Supplementary Material

Effects of inundation, nutrient availability and plant species diversity on fine root mass and morphology across a saltmarsh flooding gradient

Regine Redelstein*, Thomas Dinter, Dietrich Hertel, Christoph Leuschner

*** Correspondence:** Regine Redelstein: regine.redelstein@biologie.uni-goettingen.de

Supplementary Material 3: Principle Components Analysis (PCA) for deeper soil

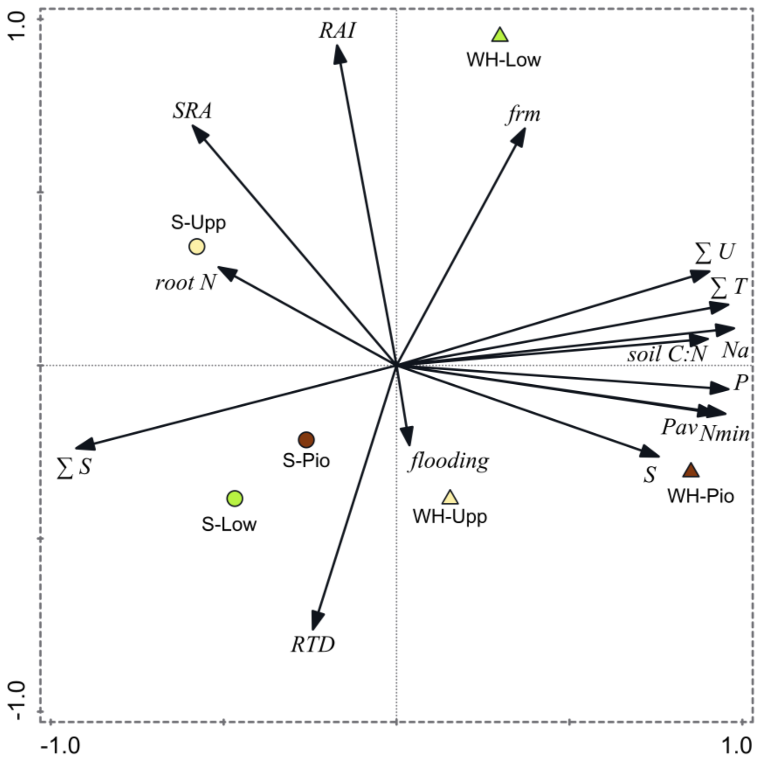

Figure S1: Plot showing the distribution of the three saltmarsh zones (Pio, Low and Upp) in the two saltmarsh sites Spiekeroog (S) and Westerhever (WH) in PCA axes 1 and 2 with soil and root properties (SRA: specific root area, ∑T: clay fraction, RAI: root area index, FRM: fine root mass, root N: root N concentration, ∑S: sand fraction, H: Shannon-Wiener index, Na: Na concentration in soil, flooding: monthly flooding events, P: total P in soil, Pav: plant-available P in soil, RTD: root tissue density, ∑U: silt fraction, Nmin: mineral N (NO_3^- and NH_4^+) in soil) related to the deeper soil horizon (G_o/G_r). Vector length and angle are proportional to the direction and degree of their correlation with the plot ordination scores.

Table S3: Results of a Principal Components Analysis (PCA) on the differentiation of the 3 saltmarsh zones at both study sites with respect to fine root mass, root morphological traits, species diversity and soil properties of the Go/Gr horizon. Given are the loadings of the selected variables along the four explanatory axes. Numbers in brackets below the axes indicate the eigenvalues (EV) of the axes. Numbers in bold mark the variables with closest correlation to the respective axis (cumulative fit values are given in brackets).

	**Axis 1 (EV 0.5633)**	**Axis 2 (EV 0.1873)**	**Axis 3 (EV 0.1430)**
fine root mass	0.38 (0.14)	**0.78** (0.61)	0.55 (0.91)
specific root area	-0.59 (0.35)	**0.69** (0.83)	-0.30 (0.92)
root tissue density	-0.24 (0.06)	**-0.76** (0.64)	-0.28 (0.72)
root N concentration	-0.51 (0.26)	0.28 (0.35)	-0.32 (0.45)
root surface area	-0.17 (0.03)	**0.92** (0.88)	0.24 (0.94)
total Na in soil	**0.98** (0.95)	0.11 (0.96)	0.05 (0.97)
total P in soil	**0.96** (0.92)	-0.07 (0.93)	-0.10 (0.94)
total S in soil	**0.76** (0.57)	-0.26 (0.64)	**0.59** (0.99)
C:N ratio in soil	**0.90** (0.81)	0.08 (0.82)	-0.13 (0.83)
plant available P	**0.92** (0.85)	-0.14 (0.87)	-0.12 (0.89)
plant available N	**0.95** (0.90)	-0.14 (0.92)	-0.11 (0.93)
∑ Sand	**-0.93** (0.86)	-0.24 (0.92)	0.25 (0.97)
∑ Silt	**0.90** (0.82)	0.27 (0.89)	-0.27 (0.96)
∑ Clay	**0.96** (0.92)	0.18 (0.95)	-0.20 (0.99)
flooding	0.04 (0.00)	-0.23 (0.05)	**0.97** (0.99)
