# Supplementary material for: Effects of Inundation, Nutrient Availability and Plant Species Diversity on Fine Root Mass and Morphology Across a Saltmarsh Flooding Gradient
Source: Front Plant Sci. 2018 Feb 6;9:98. doi: 10.3389/fpls.2018.00098 (PMC5808222; doi:10.3389/fpls.2018.00098)
Supplement: Supplementary file 4 [file DataSheet2.pdf]

Supplementary Material

Effects of inundation, nutrient availability and plant species diversity on fine root mass and morphology across a saltmarsh flooding gradient

Regine Redelstein*, Thomas Dinter, Dietrich Hertel, Christoph Leuschner

*** Correspondence:** Regine Redelstein: regine.redelstein@biologie.uni-goettingen.de

Supplementary Material 4: Data on above- and below-ground biomass at two saltmarsh sites (R. Strieckmann 1989, unpubl. data)

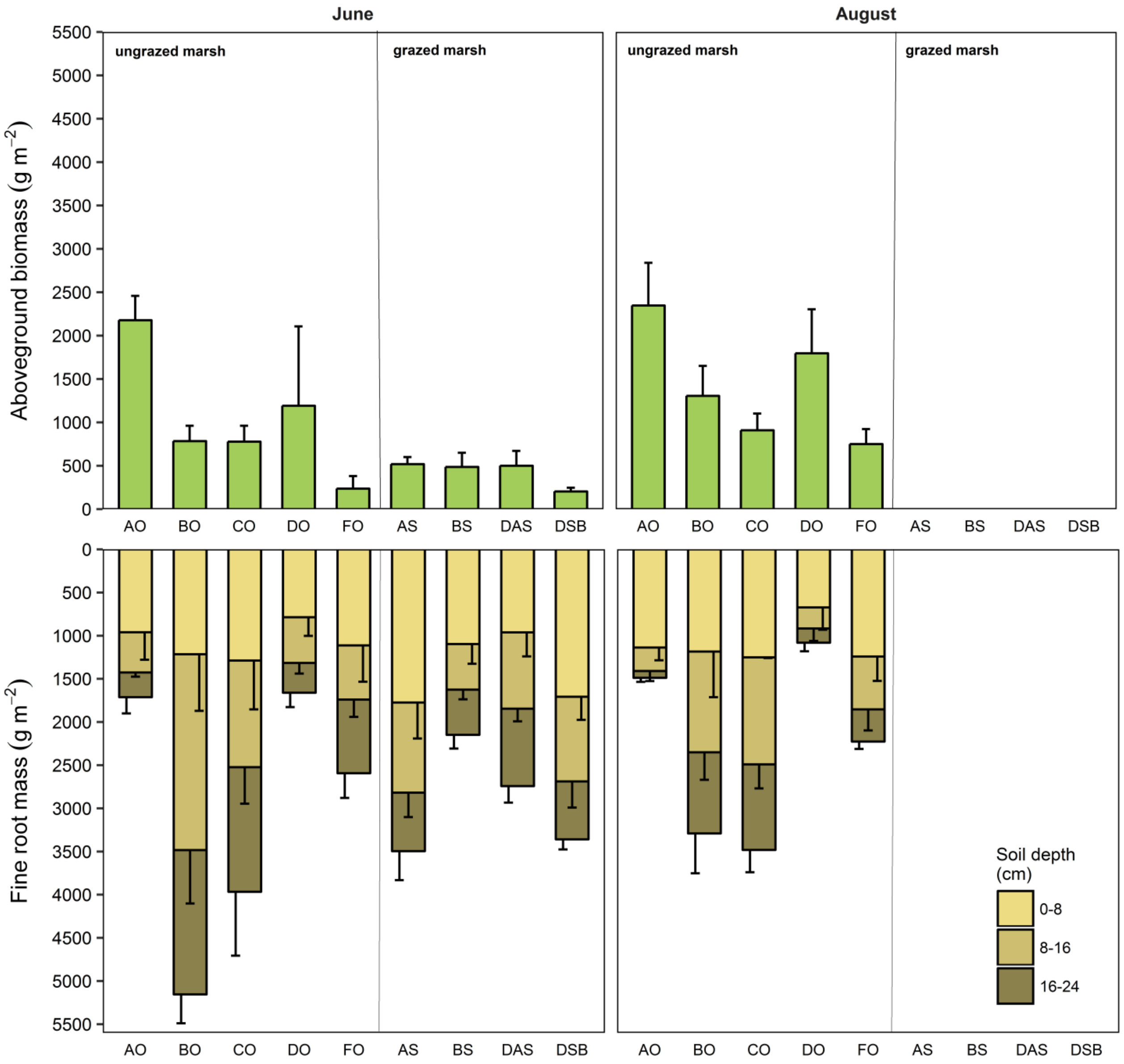

Figure S2: Above-ground vs. below-ground (fine root) biomass (dry weight) at nine plots in two saltmarsh sites (means ± SD, n = 4-6). Plot names with an "O" are located in an ungrazed salt marsh on the Hallig island Oland, plot names with an "S" are located in a grazed salt marsh at Sönke-Nissen-Koog (both are located in Schlswig-Holstein, Germany). The first letter of the plot name refers to the plant community: "AO": *Elytrigia atherica* dominated (Oland), "BO": *Spartina anglica* with *Aster tripolium* (Oland), "CO": *Spartina anglica* dominated Oland, "DO": *Atriplex portulacoides* dominated (Oland), "FO": *Spartina anglica* dominated Oland, "AS" *Spartina anglica* dominated (Sönke-Nissen-Koog), "BS": Puccinellia maritima dominated (Sönke-Nissen-Koog), "DAS": Spartina anglica dominated at a scoured location (Sönke-Nissen-Koog), "DSB": *Puccinellia maritima* dominated on an elevated location (Sönke-Nissen-Koog). Data collected by Rita Strieckmann (1989, unpublished).
